# Specific media literacy tips improve AI-generated visual misinformation discernment

**DOI:** 10.1186/s41235-025-00648-z

**Published:** 2025-07-03

**Authors:** Sean Guo, Briony Swire-Thompson, Xiaoqing Hu

**Affiliations:** 1 Department of Psychology, The University of Hong Kong, Pokfulam, Hong Kong SAR China; 2 Department of Political Science, Northeastern University, Boston, MA USA; 3 The State Key Laboratory of Brain and Cognitive Sciences, The University of Hong Kong, Pokfulam, Hong Kong SAR China; 4 HKU-Shenzhen Institute of Research and Innovation, Shenzhen, China

**Keywords:** Media literacy, Visual misinformation, AI-generated content, Misinformation discernment

## Abstract

Images generated using artificial intelligence (AI) have become increasingly realistic, sparking discussions and fears about an impending “infodemic” where we can no longer trust what we see on the internet. In this preregistered study, we examine whether providing specific media literacy tips about how to spot AI-generated images can reduce susceptibility to AI-generated visual misinformation (AIVM). Participants were randomly assigned to one of three conditions, reading specific media literacy tips, general media literacy tips, or no media literacy tips (control). The general tips provided tips on how to spot misinformation, while the specific tips provided more detailed tips for how to detect AIVM. Results showed that specific tips increased headline discernment between true and false information more than general tips. Both media literacy interventions reduced belief in AIVM compared to control, but specific tips reduced belief in AIVM more than general tips. Finally, both specific and general tips also reduced belief in real headlines compared to control, with no difference between them. In an information environment that sees increasing prevalence of AIVM, it may be worth being specific about how to detect misinformation online rather than only providing general information.

**Supplementary Information:**

The online version contains supplementary material available at 10.1186/s41235-025-00648-z.

## Specific media literacy tips improve AI-generated visual misinformation discernment

Recent advances in artificial intelligence (AI) technology have generated a great deal of discussion about their benefits and disadvantages to society. With widely available AI image generators such as Stable Diffusion (Stable Diffusion, 2024) and Midjourney (Midjourney, 2024), users can transform a text prompt to a realistic visual representation in seconds. While these technologies could certainly expedite graphic design and inspire artists, they also assist the proliferation of AI-generated visual misinformation (AIVM), harming truthful discourse (Chesney & Citron, 2019; Hameleers & Marquart, 2023; Yang et al., 2023). Although image manipulation tools have existed for decades, AI has lowered the barriers such that people with limited skills can generate convincing fake images. Indeed, posts containing AI-generated images on X (formerly Twitter) have seen a sharp increase in recent years (Corsi et al., 2024). For instance, several instances of AI-generated political disinformation and synthesized videos of politicians in the U.S., India, Pakistan and Indonesia have been reported in 2024 (Adam, 2024; Shukla & Tripathi, 2024). How to assist people to identify AIVM has thus become an essential question.

Improving media literacy, defined as “the ability to access, analyze, evaluate and create messages across a variety of contexts” (Livingstone, 2004, p.18), is a potential avenue to reduce belief in AIVM. Previous research has found that media literacy tips can decrease belief in false headlines (Clayton et al., 2020; Lutzke et al., 2019) and improve discernment between true and false news (Fazio et al., 2024; Guess et al., 2020). For example, Guess et al. (2020) found that reading tips describing how to identify fake news (i.e., investigating the source) and verification strategies (i.e., checking the evidence) significantly improved headline accuracy discernment. Similarly, Lutzke et al. (2019) found that reading guidelines on evaluating news credibility lowered trust in false headlines. Preliminary evidence in an unpublished manuscript suggests that media literacy interventions may even outperform other types of misinformation interventions in improving accuracy discernment (Fazio et al., 2024). However, the vast majority of media literacy interventions have been conducted using textual information.

An important aspect to consider when distributing media literacy tips is their level of specificity (i.e., how detailed or relevant they are to the misinformation type). Social media platforms such as Facebook once provided media literacy tips, although they remained fairly general. For instance, they provided recommendations such as being skeptical of information or paying attention to how information makes you feel (Facebook Help Center, 2019). It is possible that specific tips about a type or modality of misinformation (e.g., AIVM) could be more beneficial for detecting that type of misinformation. Research suggests that reading tips about a specific topic (i.e., misinformation on the war in Ukraine) is more effective at decreasing misinformation belief for that topic compared to general tips, if political ideology and media trust are accounted for (Hameleers & van der Meer, 2023). Furthermore, Nightingale et al. (2022) found that watching a short video on the characteristics of digitally altered photos such as airbrushing and adding/removing objects improved detection of these manipulated photos. Importantly, participants who used certain specific strategies (e.g., identifying photometric inconsistencies) were more accurate at identifying manipulated images. This evidence suggests that specific media literacy tips may be more effective than general media literacy tips at reducing belief in AIVM. However, findings have not consistently supported the superiority of specific tips. Hwang et al. (2021) provided participants with either specific tips about deepfakes or general tips about disinformation. They found that while both types of tips were effective at reducing the credibility of a deepfake news article, there was no significant difference between them. Therefore, when it comes to identifying manipulations in visual media, solid conclusions about the effectiveness of specific tips cannot be made.

Beyond media literacy tips, the specificity of warnings has been shown to play an important role in identifying and correcting misinformation. For example, specific warnings are more effective than general warnings at reducing the post-event misinformation effect (for a meta-analysis, see Blank & Launay, 2014). This effect occurs when misinformation influences participants’ memory of an event they initially witnessed (e.g., when a leading question influences recall after observing an accident; Loftus et al., 1978). Warnings about the presence of misinformation reduced participants’ memory errors about the original events, and warnings that specifically identified the misleading details were more effective than warnings that did not (Blank & Launay, 2014; Higham et al., 2017). Moreover, warnings with explanations about the misinformation effect are more effective than warnings without such an explanation (Blank & Launay, 2014). This could be because explanations challenged participants’ assumptions that post-event information is consistent with what they originally witnessed, thereby changing how they interpreted the task and improving resistance to misinformation (Blank, 1998; Oeberst & Blank, 2012). With regards to images accompanying news headlines, people may assume that the images represent reality (Messaris & Abraham, 2001; Sundar et al., 2021). Specific media literacy tips could challenge this assumption and provide details about the properties of AI-generated images, prompting people to pay more attention to image details and spend more time evaluating the headlines. Detecting details consistent with AIVM may also decrease the credibility of the headline, or boost credibility when no AIVM-related details are detected. Therefore, relative to general tips, specific tips may decrease belief in AIVM and increase belief in real headlines.

The benefits of specific warnings over general warnings have also been examined in the context of the continued influence effect (CIE), a phenomenon in which misinformation continues to influence subsequent memory, attitudes and behavior after correction (Ecker et al., 2022; Wilkes & Leatherbarrow, 1988). While general warnings (i.e., telling participants that information is not always accurate) can reduce the CIE, specific warnings (i.e., providing a detailed description of the CIE and how it is elicited) are even more effective (Ecker et al., 2010). Ecker et al. speculated that specific warnings led participants to better identify misinformation during encoding, improving the effectiveness of subsequent corrections. Specific tips about AIVM may similarly improve AIVM identification in our study.

The primary aim of this preregistered study was to examine whether specific tips decrease belief in AIVM more than general tips, and whether both types of tips decrease belief in AIVM more than a control condition. In addition, we aimed to examine how specific and general tips influenced belief in real headlines, given findings that media literacy tips could also decrease belief in true information (Hoes et al., 2024; van der Meer et al., 2023). We also conducted analyses on discernment and response time when rating headline accuracy, and non-preregistered linear mixed model analyses correcting for random effects of headline and participant. We hypothesized that (a) specific tips would decrease belief in AIVM the most when compared to general tips or control, (b) both general and specific tips would decrease belief in real headlines compared to control, and (c) specific tips would not decrease belief in real headlines as much as general tips, given that specific tips provide a more targeted intervention that impacts AIVM alone (see Supplement A for all preregistered hypotheses and whether they were supported or rejected).

## Method

### Sample size justification

Previous studies of media literacy interventions found small effects (*d* = 0.08: Clayton et al., 2020; *d* = 0.11, 0.20: Guess et al., 2020). We thus aimed to detect a small effect size. Using G*Power 3.1 with f = 0.10, a minimum sample size of 323 per condition was required to achieve 80% power (Erdfelder et al., 2009). We therefore aimed for a total sample of 969 people.

### Participants

We recruited 1027 individuals from Prolific. Fifteen participants were excluded because they indicated in an honesty check question that they had not put their best effort into their responses (n = 3), submitted the survey multiple times (n = 3), or provided identical responses to over 80% of ratings (n = 9). In the final analyses, we included 1012 participants (*M*_age_ = 41.9, *SD* = 14.1, ranged from 18 to 95 years old, 462 males and 539 females, and 11 who did not report their gender). Among these participants, 333 were in the specific tips condition, 341 were in the general tips condition, and 338 were in the no tips condition. Participants currently resided in the U.S., spoke English as their first language, and had at an approval rating of at least 90% on Prolific. This research was approved by the Human Research Ethics Committee of the University of Hong Kong (EA210341). Participants provided consent prior to participation.

### Procedure

Participants were randomized into one of three conditions: specific media literacy tips, general media literacy tips, and no tips (i.e., control). In the specific tips condition, participants read a paragraph about AI-generated misinformation, followed by tips on how to spot AI-generated images. These were specific recommendations regarding how to detect AI-generated images, and contained three tips: identify abnormal details, check for incoherent text, and be suspicious of images with a crisp foreground and blurry background, as seen in Table 1. In the general tips condition, participants read a paragraph about misinformation in general, followed by three tips adapted from Facebook’s tips to detect fake news (Facebook Help Center, 2019). These entailed being skeptical of information, paying attention to how information makes you feel, and checking if images are authentic, as seen in Table 1. For both general and specific tips, each tip was accompanied by a relevant image and a short blurb expanding on the tip (see Supplement B for full details). Specific and general tips were of similar length (197 vs. 195 words). Participants in the control condition moved directly to the headline rating task.

**Table 1:** Specific and general tips

Specific Tips	Tip Image
1. Abnormal details Many AI-generated images look convincing, but closer inspection often reveals inconsistent or strange details. For example, this image of monks in a brawl contains hands with more than five fingers, and strangely positioned limbs. These incorrect details are often situated in the background and are more difficult to detect	
2. Incoherent text AI often struggles to create coherent text. Keep an eye out on any strange text (including on signs, clothing or books) to identify if an image may be AI-generated. Text may look convincing at a glance, but is often nonsensical or misspelled	
3. Sharp foreground, blurry background Images created by AI often have crisp and clear foregrounds or subjects with a blurry background, often to hide incoherent details	

General Tips	Tip Image
1. Be skeptical of information False information tends to be exaggerated and is often written to be catchy and sensational. If something sounds too unbelievable to be true, it often is	
2. Pay attention to how information makes you feel False information often uses emotionally charged language to manipulate your feelings and incite anger, fear, excitement, or other strong emotions. This is a tactic to make you more likely to share the content without questioning its validity. Take a moment to critically evaluate the information before accepting it as truth	
3. Check if images are authentic False information may sometimes be accompanied by doctored images or images taken out of context to support their claims. Be on the lookout for these, as they may signal an untrustworthy source	

Tips presented in the Specific and General Conditions. The control condition did not receive any tips. Refer to Supplement B for the formatted tips including a short introductory paragraph on AIVM (specific tips condition) and general misinformation (general tips condition)

**Fig. 1:**
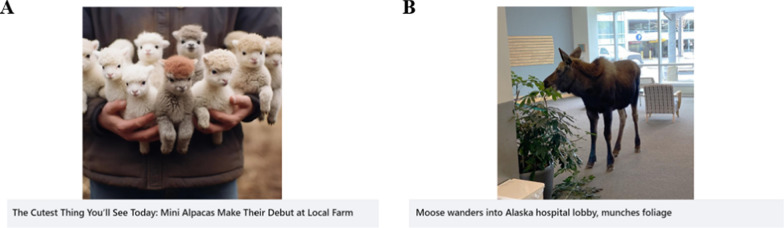
Example headlines. **A** Example AIVM headline with an AI-generated image and **B** example real headline with a real image

Participants then completed the headline rating task. We used 20 real headlines with real images, and 20 false headlines with AI-generated images from Guo et al., (2024) for the task. These covered a wide range of topics including accidents, funny stories, and strange phenomena, but did not include any political or health-related headlines to minimize effects of prior attitudes on belief (see Fig. 1 for example headlines). No sources or engagement metrics were provided to participants. Participants viewed each headline in a randomized order and rated their belief in the headline on a scale from 0 (definitely false) to 10 (definitely true). Two attention checks were given randomly during this task to ensure attentiveness. Attention check headlines included a real image that was not used in any other headline, and explicitly instructed participants to provide a specific rating (“0”, or “10”) on the belief scale.

After the headline rating task, participants who read specific tips answered one question about each of the three specific tips to examine how well they remembered the tips (Supplement B.2). Finally, all participants answered an honesty check question, were asked about their demographics, and were debriefed as to which headlines were real and false.

## Results

Participants did not differ significantly in age (*p* = 0.784), education (*p* = 0.455), or gender (*p* = 0.332) across conditions. We report results that exclude participants (*n* = 27) who failed both attention checks during the headline rating task. However, results did not differ from the full sample of 1039 participants (Supplement C). Our goal was to measure differences between specific and general tips conditions with regards to AI-generated visual misinformation (AIVM) belief, real headline belief, discernment, and belief rating response time.^1^ For all Bayesian analyses, the null hypothesis (H_0_) was modeled as a point null, and the alternative hypothesis (H_1_) was specified using a two-tailed Cauchy prior distribution centered at zero with a scale parameter of 0.707 (Rouder et al., 2009).

### Belief in AIVM

We first examined whether belief in AIVM differed between tip conditions by conducting a one-way Welch’s ANOVA with belief in AIVM as the outcome measure and found significant differences between conditions (*F*(2, 668) = 24.81, *p* < 0.001, η_p_^2^ = 0.07). Games-Howell post hoc tests revealed that the control condition (*M* = 4.35, *SE* = 0.07) had higher belief in AIVM compared to participants exposed to general tips (*M* = 3.84, *SE* = 0.07), *t*(665) = 5.10, *p* < 0.001, *d* = 0.38, and specific tips (*M* = 3.62, *SE* = 0.08), *t*(668) = 6.81, *p* < 0.001, *d* = 0.55. Participants exposed to specific tips had numerically lower belief in AIVM than those exposed to general tips, but the difference was not significant, *t*(652) = 2.22, *p* = 0.069, *d* = 0.17. A follow-up Bayesian analysis provides almost equal evidence (BF_01_ = 1.04) favoring either the null (i.e., no difference between specific and general tips) or the alternative (i.e., a range of possible differences in AIVM belief between specific and general tips, centered on zero) hypotheses.

### Belief in real headlines

**Fig. 2:**
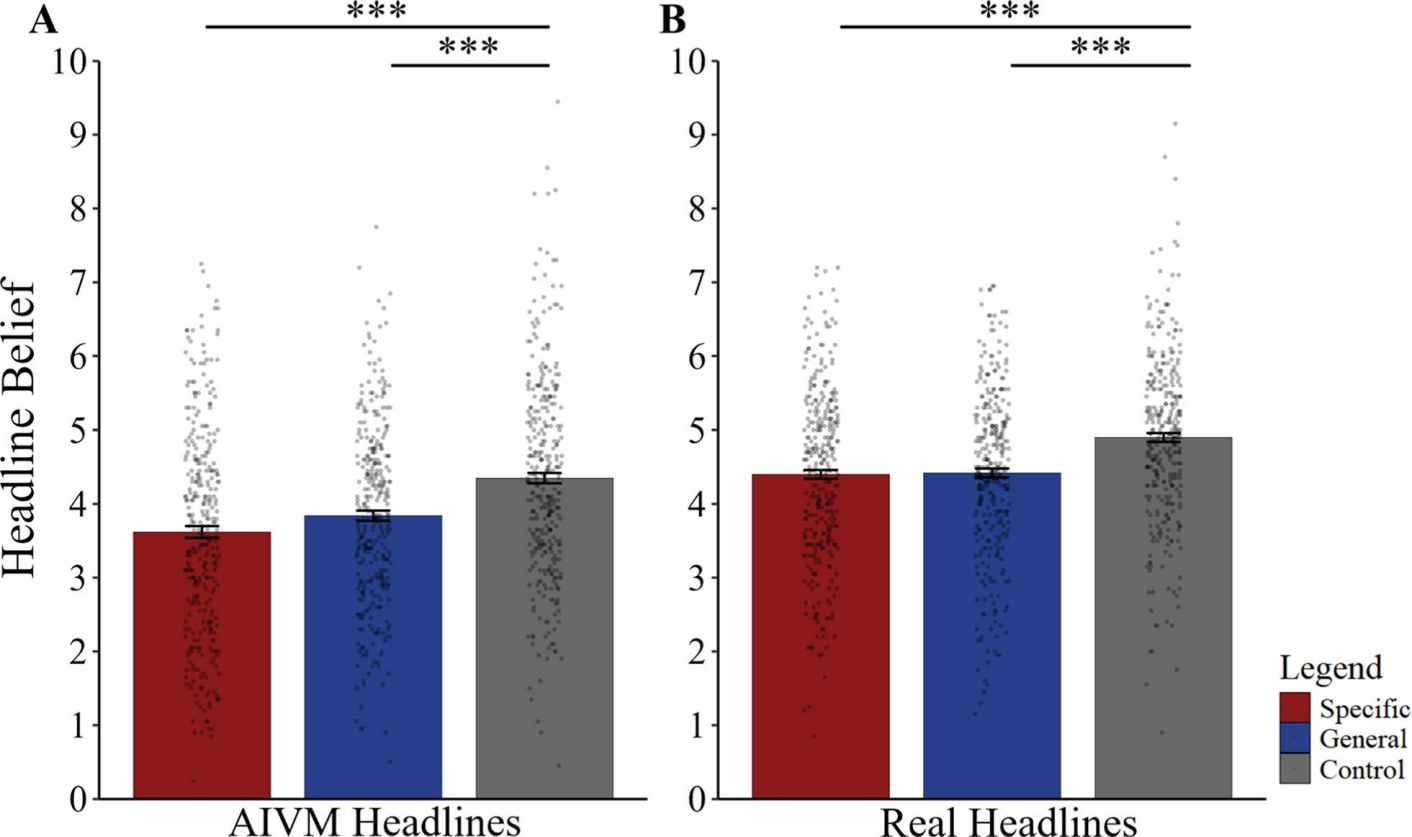
Belief in AIVM and real headlines in specific tips, general tips and control conditions. Belief in **A** AIVM headlines & **B** Real headlines in specific, general and no tips conditions. Error bars indicate standard error

Next, we examined whether belief in real headlines differed between tip conditions using a one-way Welch’s ANOVA with belief in real headlines as the outcome measure. There were significant differences between conditions (*F*(2, 672) = 20.86, *p* < 0.001, η_p_^2^ = 0.06), and Games-Howell post hoc tests revealed that the control condition (*M* = 4.90, *SE* = 0.06) had higher belief in real headlines compared to participants who read the general tips (*M* = 4.42, *SE* = 0.06), *t*(676) = 5.54, *p* < 0.001, *d* = 0.42, and specific tips (*M* = 4.40, *SE* = 0.06), *t*(668) = 5.68, *p* < 0.001, *d* = 0.44. However, participants who read the specific and general tips did not significantly differ, *t*(668) = 0.28, *p* = 0.957, *d* = 0.02. A follow-up Bayesian analysis showed strong evidence favoring the null (i.e., no difference between specific and general tips) hypothesis (BF_01_ = 11.20) (Fig. 2).

### Discernment

Next, we conducted pre-registered discernment analyses. Discernment (d’) was calculated as z(proportion of hits)—z(proportion of false alarms), with hits and false alarms defined as ratings of six to ten for real headlines and AIVM respectively. An examination of a Q–Q plot of residuals revealed that the data was not normally distributed. Therefore, we opted to deviate from our preregistration and conduct a Kruskal–Wallis test. Results revealed significant differences between conditions, χ^2^(2) = 13.35, *p* = 0.001, with median d’ of 0.40 in the specific tips condition, 0.30 in the general tips condition, and 0.25 in the control condition. Wilcox rank-sum post-hoc tests showed that participants in the specific tips condition had higher d’ than those in the general tips (W = 63,526, *p* = 0.011, *r* = 0.103) and control (W = 64,976, *p* = 0.001, *r* = 0.134) conditions. Participants in the control condition did not significantly differ in d’ than those in the general tips condition (W = 60,128, *p* = 0.328, *r* = 0.037). These results showed that discernment was highest for those in the specific tips condition, and discernment did not significantly differ for those in general tips and control conditions. See table S3 for d’, response bias, false alarm and hit rate descriptives.

**Fig. 3:**
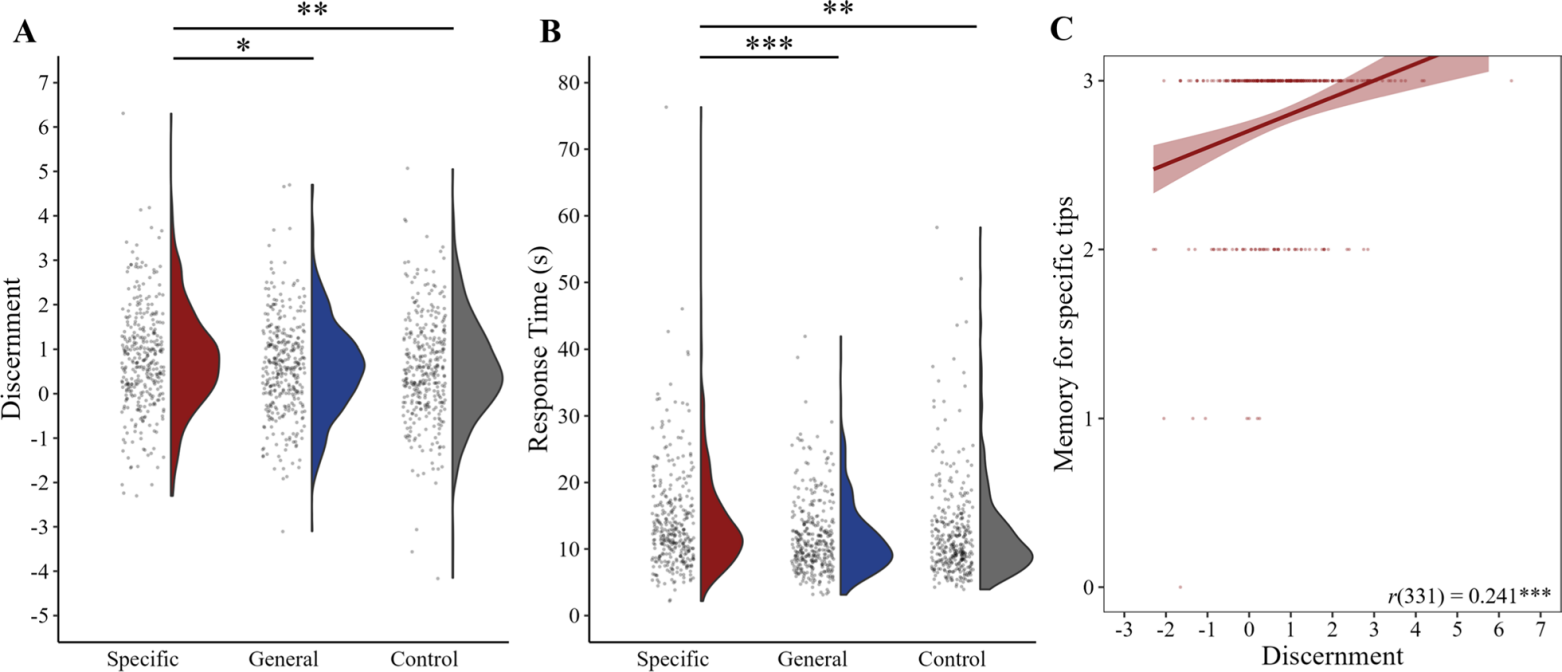
Discernment (difference score), response time and memory for specific tips. Violin plot showing **A** discernment (difference score) between AIVM and real headlines, and **B** average response times for belief ratings in specific, general and no tips conditions. **C** Correlation between discernment (difference score) and memory for specific tips. Shaded area represents 95% confidence interval

Although d’ may be a useful measure to determine discernment between real and false headlines (Gawronski et al., 2023, 2024), other studies have suggested that d’ is reliable when there are many trials (e.g., 140 headlines in Dobbs et al., 2023, 400 trials in Guggenmos, 2021). Therefore, the 40 headlines used in our study may not provide a reliable estimate of d’. Additionally, the d’ measure collapsed ratings on the belief scale to binary responses (ratings less than 5 were “false”, while ratings greater than 5 were “true”), which may obscure the extent of headline beliefs. Therefore, we conducted non-preregistered analyses using participant-level difference scores, calculated as belief in real headlines minus belief in AIVM headlines. An examination of residuals revealed that the data was not normally distributed, and thus we opted to conduct a Kruskal–Wallis test. Results revealed significant differences between conditions, χ^2^(2) = 7.40, *p* = 0.024, with median difference score of 0.70 in the specific tips condition, 0.60 in the general tips condition, and 0.50 in the control condition, as seen in Fig. 3A. Wilcox rank-sum post-hoc tests showed that participants in the specific tips condition had greater difference scores than those in the general tips (W = 51,363, *p* = 0.048, *r* = 0.082) and control conditions (W = 49,946, *p* = 0.034, *r* = 0.097). Difference scores did not significantly differ between participants in the control condition and those in the general tips condition (W = 56,564, *p* = 0.677, *r* = 0.016). Post-hoc tests were corrected for multiple comparisons by the FDR method. These results showed that participants in the specific tips condition showed a larger difference in beliefs between AIVM headlines and real headlines compared to those in the general tips and control conditions.

### Response time

Next, we examined how specific and general tips affected belief rating response time, as preregistered. An examination of a Q–Q plot of residuals revealed that the data was not normally distributed. Therefore, we opted to deviate from our preregistration and conduct a Kruskal–Wallis test. Results revealed significant differences between conditions, χ^2^(2) = 28.78, *p* < 0.001, with median RT of 12.28 s in the specific tips condition, 10.52 s in the general tips condition, and 10.34 s in the control condition, as seen in Fig. 3B. Wilcox rank-sum post-hoc tests showed that participants in the specific tips condition had higher RT than those in the general tips and control conditions, W = 68,942, *p* < 0.001, *r* = 0.185, W = 67,523, *p* < 0.001, *r* = 0.173 respectively. Participants in the control condition did not significantly differ in response time than those in the general tips condition (W = 57,076, *p* = 0.829, *r* = 0.008). Post-hoc tests were corrected for multiple comparisons by the FDR method. In sum, results show that participants in the specific tips condition spent the most time rating headlines compared to general tips and control conditions.

### Linear mixed-model analyses (Not preregistered)

To better account for within-participant variation and differences in headline characteristics (as in Clayton et al., 2020; Fazio et al., 2024; Guess et al., 2020), we conducted exploratory linear mixed model analyses using the lme4 package in R (Bates, 2005; Bates et al., 2015). The dependent variable was headline belief, and we included fixed effects of condition (specific, general and control), headline veracity (real, AIVM), and their interaction. Random intercepts of participant and headline were included. Because we were particularly interested in seeing how the specific tip condition compared to other conditions, we set the specific tip condition as the reference level. We used Satterthwaite’s approximation to calculate p-values with the lmertest package (Kuznetsova et al., 2017, R Core Team, 2022).

We found that there was a significant positive interaction between the general tips condition and belief in AIVM headlines (*b* = 0.20, *SE* = 0.06, *p* < 0.001) and between the control condition and AIVM headlines (*b* = 0.23, *SE* = 0.06, *p* < 0.001). This suggested that compared to participants in the specific tip condition, participants in both the general tips and control conditions showed less difference in belief between AIVM and real headlines (i.e., decreased discernment). Post hoc tests revealed that for AIVM, participants in the specific tips condition showed less belief compared to those in the general tips condition with an estimate of 0.22 (z = 2.49, *SE* = 0.09, *p* = 0.013). Participants in the control condition had significantly higher belief in AIVM than those in the general tips condition with an estimate of 0.51 (z = 5.63, *SE* = 0.09, *p* < 0.001), and had significantly higher belief in AIVM than those in the specific tips condition with an estimate of 0.73 (z = 8.09, *SE* = 0.09, *p* < 0.001). For real headlines, belief did not differ between participants in the specific tips condition and those in the general tips condition, with an estimate of 0.02 (z = 0.28, *SE* = 0.09, *p* = 1.0). Participants in the control condition had significantly higher belief in real headlines than those in the general tips condition with an estimate of 0.48 (z = 5.33, *SE* = 0.09, *p* < 0.001) and those in the specific tips condition with an estimate of 0.50 (z = 5.57, *SE* = 0.09, *p* < 0.001). These results suggest that when accounting for random intercept effects of participants and headlines, specific tips exhibited higher discernment and lower belief in AIVM headlines compared to the general tips and control conditions, while belief in real headlines did not differ between specific and general tips. Post-hoc tests were corrected for multiple comparisons by the false discovery rate (FDR) method. See table S5 for full model statistics.

### Correlation between memory for specific tips and discernment (Not preregistered)

Within the specific tips condition, memory for tips was positively correlated with d’, *r*(331) = 0.239, *p* < 0.001, indicating that improved memory for tips was associated with greater discernment between real and AIVM headlines, as seen in Fig. 3C. Memory for tips was also positively correlated with the difference score, *r*(331) = 0.241, *p* < 0.001, indicating that the difference in beliefs between real and AIVM headlines was greater when more tips were remembered.

## Discussion

In the current study, we examined the effects of a media literacy intervention on belief in AI-generated visual misinformation (AIVM). We provided participants with either specific media literacy tips on how to identify AIVM, general media literacy tips on how to identify false information, or no media literacy tips (a control). We measured belief in AIVM, real headlines, discernment between the two, and response times during these ratings. We found that both types of tips decreased belief in AIVM compared to control, and specific tips decreased belief in AIVM more than general tips when the variation within participants and between headlines was accounted for. Providing specific tips boosted discernment between real headlines and AIVM more than general tips, and specific tips encouraged people to spend more time evaluating headlines, potentially allowing them the opportunity to engage further with the images.

Our prediction that reading specific tips would decrease belief in AIVM headlines and increase belief in real headlines was partially supported: those in the specific tips condition had the highest discernment, and lowest belief in AIVM headlines. However, belief in real headlines was the same between specific and general tip conditions. This suggests that the improved discernment was primarily driven by decreased AIVM headline belief. Research on the post-event misinformation effect has shown that specific warnings are effective because they challenge implicit assumptions that subsequent misinformation is consistent with the original event, potentially promoting conflict detection between original and post-event information (Blank, 1998; Oeberst & Blank, 2012). Warned participants also engage in more effortful monitoring processes during retrieval and spend more time thinking about their responses (Echterhoff et al., 2005). Similarly, reading specific tips may prompt people to pay more attention to images by challenging their assumptions about visual evidence (Messaris & Abraham, 2001; Sundar, 2008). Indeed, people who read specific tips about AI-generated images and their characteristics took longer to rate their beliefs, potentially reflecting more analytical processing of headline images to identify flaws. To our knowledge, this is the first evidence showing how a media literacy intervention changes the speed of headline belief ratings, and integrating this measure into future studies may contribute to understanding the mechanisms of why specific tips improve AIVM discernment.

It is important to note that the differences in AIVM headline belief between specific and general tip conditions were only statistically significant when we accounted for random intercept effects of headline and participant. This suggests that there was variability in headline believability and participant responses that could only be captured with a regression model. Although this analysis was not preregistered, it is similar to analysis methods used in previous media literacy intervention studies (Clayton et al., 2020; Fazio et al., 2024; Guess et al., 2020).

Like our study, Hameleers and van der Meer (2023) found that reading tips about a specific topic was more effective at decreasing misinformation belief compared to reading general tips. Unlike our study, specific tips in their study did not improve overall discernment, which suggests that the relationship between specific tips and discernment may be topic dependent. Our findings also contradict Hwang et al. (2021), who found that specific and general tips were equivalent for reducing people’s beliefs in deepfake materials. Deepfakes primarily alter human faces from existing videos (Sharma & Kaur, 2022), potentially rendering specific tips less effective due to increased difficulty in detection. Indeed, other studies have shown that reading about characteristics of deepfakes does not improve their detection (Bray et al., 2023; Somoray & Miller, 2023). Overall, the effectiveness of specific tips may vary according to topic or news stimuli, consistent with the idea that effects of media literacy interventions vary based on context and audience characteristics (Tully et al., 2020; van der Meer et al., 2023).

A common side effect of media literacy interventions is that belief in real information is reduced because of increased skepticism (Hoes et al., 2024; van der Meer et al., 2023). We found that both types of tips indeed decreased belief in real headlines compared to control, with Bayesian analyses providing strong evidence that there was no difference between the two. Although it is unfortunate that the media literacy tips led to lower belief in real information, at least the specific tips did not increase distrust in facts compared to general literacy tips (see Schiff et al., 2023 for an alternate view). Furthermore, reading specific tips improved discernment compared to the control condition while reading general tips did not. Whereas previous studies found that general tips improved discernment (Fazio et al., 2024; Guess et al., 2020), these studies primarily investigated textual misinformation. Discerning between AIVM and real headlines may thus require more specific tips than textual information.

As an exploratory analysis, we found that memory for specific tips was positively correlated with discernment, suggesting that the benefits of the tips may be greater for those who remember them. Similarly, a previous study showed that participants who correctly answered questions about media literacy tips were better able to differentiate between real and false news headlines (Guess et al., 2020). Therefore, making tips memorable by using mnemonic techniques (e.g., rhymes, acronyms, associations) could potentially improve their effectiveness. However, it remains unclear whether memory plays an equally important role for both specific and general tips, as we did not measure memory for general tips in our study.

This study has several limitations. Specific tips about AIVM may quickly become obsolete due to the ever-changing landscape of AI, which may pose challenges to implementing real-world media literacy interventions. For example, some AI image generators can already generate coherent text within images. To address this, tips may need to be frequently updated. Specific tips may also only improve detection of AIVM with the characteristics mentioned in the tips (i.e., abnormal details, incoherent text, blurry backgrounds) but not for AIVM with other characteristics. We note that 50% of the AIVM in our study had at least one of the features mentioned in the specific tips. Thus, whether these specific tips can generalize to AIVM with other characteristics should be further investigated. Finally, our finding that better memory for specific tips correlated with greater discernment should be interpreted with caution, as it is possible that having a better memory may be linked to greater general cognitive abilities such as reading comprehension and intelligence (Hultsch et al., 1990), which could also aid discernment between headlines. Future media literacy studies should thus administer memory tests in all experimental conditions to facilitate direct comparison between conditions.

To conclude, AIVM is fast becoming a problematic issue, particularly given the increasing prevalence of AI-generated media online and rapid development of AI technology. Our study shows that readers exposed to specific media literacy tips could better differentiate between AIVM and real headlines and spent more time evaluating headlines compared to readers who were given general media literacy tips. When variations within each participant and differences between headlines were controlled for, reading specific tips also led to decreased belief in AIVM. Deploying specific tips on social media platforms could assist people in discerning between real and AIVM headlines, and future research should focus on validating these findings and their long-term effectiveness.

## Supplementary Information

Additional file 1.

## Data Availability

The dataset generated during the current study is available at https://osf.io/r6z7q. This experiment was preregistered at https://osf.io/53ta9.

## Footnotes

1 As preregistered, ratings of image surprise, realism and evidence strength from a previous study (Guo et al., (2024)) were correlated with average belief in a headline across all conditions. Results are presented in Supplement D.
