# Supplementary material for: Specific media literacy tips improve AI-generated visual misinformation discernment
Source: Cogn Res Princ Implic. 2025 Jul 3;10:38. doi: 10.1186/s41235-025-00648-z (PMC12229391; doi:10.1186/s41235-025-00648-z)
Supplement: Supplementary file 1 — Additional file 1. [file 41235_2025_648_MOESM1_ESM.docx]

**Supplementary Information for**

**Specific Media Literacy Tips Improve AI-generated Visual Misinformation Discernment**

Sean Guo^1^, Briony Swire-Thompson^2^ & Xiaoqing Hu^*1, 3, 4^

^1^Department of Psychology, The University of Hong Kong, Hong Kong SAR, China

^2^ Department of Political Science, Northeastern University, Massachusetts, United States of America

^3^The State Key Laboratory of Brian and Cognitive Sciences, The University of Hong Kong, Hong Kong SAR, China

^4^HKU-Shenzhen Institute of Research and Innovation, Shenzhen, China

**Contents**

[Supplement A: Pre-registered hypotheses 3](#_Toc188618139)

[1. Table S1 3](#_Toc188618140)

[Supplement B: Experimental stimuli 4](#_Toc188618141)

[1. Specific Tips 4](#_Toc188618142)

[2. Specific Tips Recollection questions 5](#_Toc188618143)

[3. General tips 6](#_Toc188618144)

[Supplement C: Analyses including all participants 8](#_Toc188618145)

[1. Belief in AIVM 8](#_Toc188618146)

[2. Belief in real headlines 8](#_Toc188618147)

[3. Discernment 8](#_Toc188618148)

[4. Response Time 8](#_Toc188618149)

[5. Difference Score (Not preregistered) 9](#_Toc188618150)

[6. Linear Mixed-models Analysis (Not preregistered) 9](#_Toc188618151)

[7. Correlation between memory for specific tips and discernment (Not preregistered) 10](#_Toc188618152)

[Supplement D: Realism, evidence and image surprise 12](#_Toc188618153)

[Supplement E: Discernment Descriptives 13](#_Toc188618154)

[1. Table S3 13](#_Toc188618155)

[2. Table S4 13](#_Toc188618156)

[Supplement F: Linear Mixed Models Analysis 14](#_Toc188618157)

[1. Table S5 14](#_Toc188618158)

# Supplement A: Pre-registered hypotheses

## Table S1

*Original preregistered hypotheses (H) and support from final study*

| Hypothesis | Supported/Not Supported |
| --- | --- |
| H1a: Both general and detailed warning will reduce belief in AIVM more than control. | Supported |
| H1b. Detailed warnings will decrease belief in false AIVM more than general warnings. | Partially supported – belief in AIVM was lower in the detailed warning condition compared to the general warning condition when linear mixed models analysis that accounted for random effects of subject and headline was used. |
| H2a: Both general and detailed warning will reduce belief in real headlines more than control. | Supported |
| H2b: Detailed warnings will reduce belief in real headlines less than general warnings. | Not Supported |
| H3: Our past studies with the same headlines (Guo et al, 2024) have shown that more realistic images that provide strong evidence to a headline led to stronger belief in both AIVM and real headlines. We predict the same findings in this study. | Partially supported - belief in AIVM was correlated with realism but not evidence. Belief in real headlines was correlated with both realism and evidence. |

*Note*: in the final manuscript, the term ‘warning’ was changed to ‘media-literacy tips’, and the ‘detailed’ condition was renamed to the ‘specific’ condition.

# Supplement B: Experimental stimuli

## Specific Tips

The development of artificial intelligence (AI) technology has revolutionized image creation. By simply providing a descriptive phrase, anyone can create realistic depictions of fictional events.

Unfortunately, this also means that increasingly convincing misinformation can be created and distributed. AI-generated images can lend credibility to previously unbelievable claims, since people tend to rely on visual evidence to determine if a claim is true.

How can we detect these AI-generated images? Here are a few tips:

1. Abnormal details


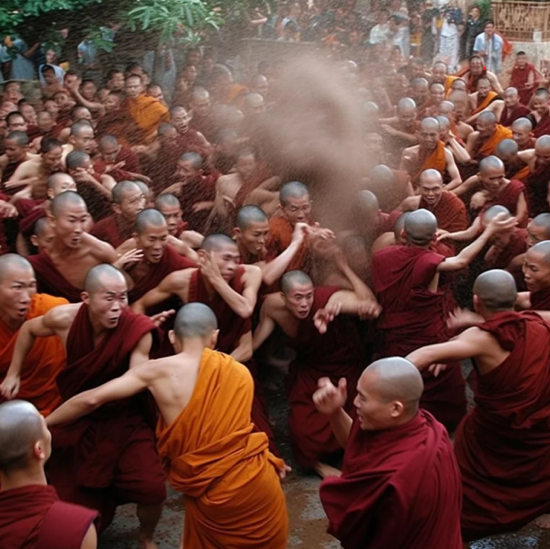


Many AI-generated images look convincing, but closer inspection often reveals inconsistent or strange details. For example, this image of monks in a brawl contains hands with more than five fingers, and strangely positioned limbs. These incorrect details are often situated in the background and are more difficult to detect.

1. Incoherent text


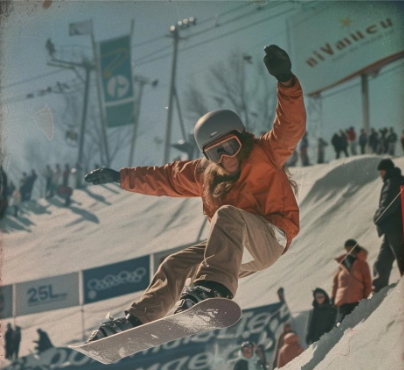


AI often struggles to create coherent text. Keep an eye out on any strange text (including on signs, clothing or books) to identify if an image may be AI-generated. Text may look convincing at a glance, but is often nonsensical or misspelled.

1. Sharp foreground, blurry background


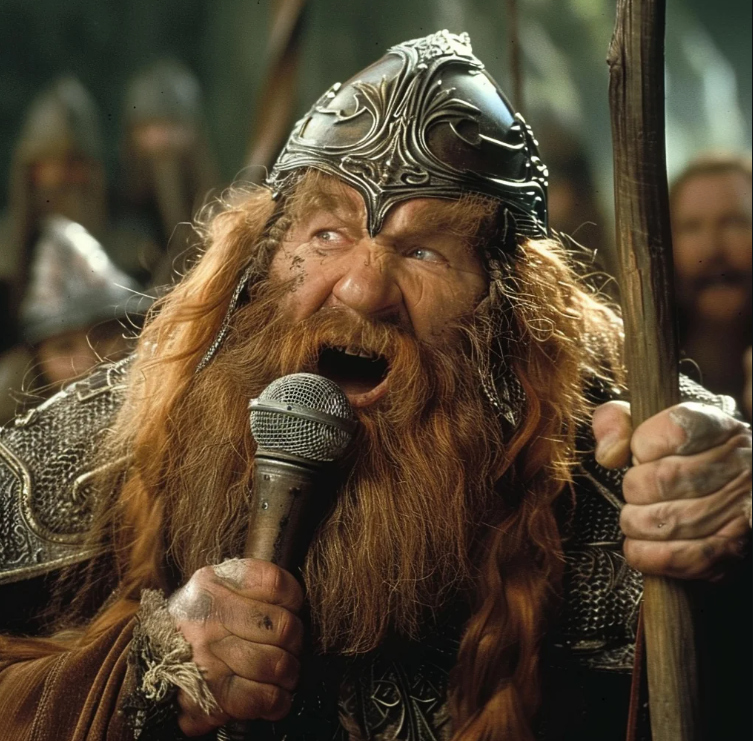


Images created by AI often have crisp and clear foregrounds or subjects with a blurry background, often to hide incoherent details.

## Specific Tips Recollection questions

1. Where are incorrect details on AI-generated images usually located?
2. Foreground
3. Background
4. AI-generated images always contain accurate text.
5. True
6. False
7. AI-generated images typically have…
8. A clear foreground and a blurry background
9. A clear background and a blurry foreground

## General tips

Misinformation has become an increasingly prevalent issue in the digital age. False information spreads quickly and easily across social media, often outpacing the dissemination of accurate information.

The distribution of false information leads to a misinformed society, which has negative implications for public health, politics, and trust in authentic information. Here are a few tips to help you remain vigilant and identify potentially inaccurate news stories online.

1. Be skeptical of information


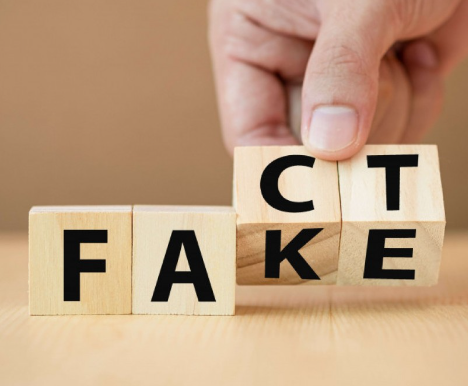


False information tends to be exaggerated and is often written to be catchy and sensational. If something sounds too unbelievable to be true, it often is.

1. Pay attention to how information makes you feel


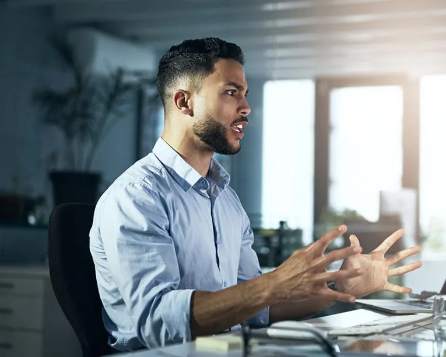


False information often uses emotionally charged language to manipulate your feelings and incite anger, fear, excitement, or other strong emotions. This is a tactic to make you more likely to share the content without questioning its validity. Take a moment to critically evaluate the information before accepting it as truth.

1. Check if images are authentic


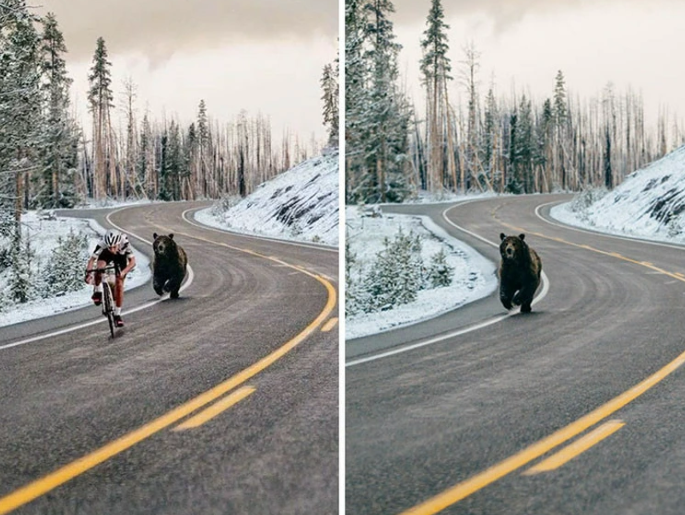


False information may sometimes be accompanied by doctored images or images taken out of context to support their claims. Be on the lookout for these, as they may signal an untrustworthy source.

# Supplement C: Analyses including all participants

## Belief in AIVM

In order to examine belief in AIVM, we first conducted a one-way Welch’s ANOVA with belief in AIVM as the outcome measure and found significant differences between conditions (*F*(2, 686) = 24.0, *p* < .001, η_p­_­­^2^ = 0.07). Games-Howell post hoc tests revealed that the control condition (*M* = 4.38, *SE* = 0.07) had higher belief in AIVM compared to participants exposed to general tips (*M* = 3.88, *SE* = 0.07), *t*(684) = 5.05, *p* < .001, *d* = 0.37, and specific tips (*M* = 3.67, *SE* = 0.08), *t*(686) = 6.68, *p* < .001, *d* = 0.53. Participants exposed to specific tips had numerically lower belief in AIVM than those exposed to general tips but the difference was not significant, *t*(670) = 2.12, *p* = .086, *d* = 0.16. Follow-up Bayesian analysis showed weak evidence favoring null hypothesis (BF_01_ = 1.30).

## Belief in real headlines

Next, we examined belief in real headlines. A one-way Welch’s ANOVA with belief in real headlines as the outcome measure showed significant differences between conditions (*F*(2, 690) = 19.7, *p* < .001, η_p­_­­^2^ = 0.05). Games-Howell post hoc tests revealed that the control condition (*M* = 4.92, *SE* = 0.06) had higher belief in real headlines compared to participants who read the general tips (*M*  = 4.45, *SE* = 0.06), *t*(693) = 5.44, *p* < .001, *d* = 0.40, and specific tips (*M* = 4.43, *SE* = 0.06), *t*(686) = 5.41, *p* < .001, *d* = 0.42. However, participants who read the specific and general tips did not significantly differ, *t*(685) = 0.18, *p* = .983, *d* = 0.01. Follow-up Bayesian analysis showed strong evidence favoring the null hypothesis (BF_01_ = 11.60).

## Discernment

Next, we conducted pre-registered discernment analyses. Discernment (d’) was calculated as z(proportion of hits) – z(proportion of false alarms), with hits and false alarms defined as ratings of six to ten for real headlines and AIVM respectively. An examination of a Q-Q plot of residuals revealed that the data were not normally distributed. Therefore, we opted to deviate from our preregistration and conduct a Kruskal-Wallis test. Results revealed significant differences between conditions, χ^2^(2) = 15.02, *p* < .001, with median d’ of 0.40 in the specific tips condition, 0.29 in the general tips condition, and 0.25 in the control condition, as seen in Figure 3A. Wilcox rank-sum post-hoc tests showed that participants in the specific tips condition had higher d’ than those in the general tips and control conditions, W = 67,324, *p* = .006, *r* = 0.108, W = 69,118, *p* < .001, *r* = 0.140 respectively. Participants in the control condition did not significantly differ in d’ than those in the general tips condition (W = 63,256, *p* = .308, *r* = 0.039). Post-hoc tests were corrected for multiple comparisons by the FDR method. These results showed that discernment was highest for those in the specific tips condition, and discernment did not significantly differ for those in general tips and control conditions.

## Response Time

To observe how specific and general tips affected belief rating response time, we conducted pre-registered analysis on response time. An examination of a Q-Q plot of residuals revealed that the data were not normally distributed. Therefore, we opted to deviate from our preregistration and conduct a Kruskal-Wallis test. Results revealed significant differences between conditions, χ^2^(2) = 32.0, *p* < .001, with median RT of 12.34s in the specific tips condition, 10.49s in the general tips condition, and 10.25s in the control condition, as seen in Figure 3B. Wilcox rank-sum post-hoc tests showed that participants in the specific tips condition had higher RT than those in the general tips and control conditions, W = 73,188, *p* < .001, *r* = 0.193, W = 71,877, *p* < .001, *r* = 0.180 respectively. Participants in the control condition did not significantly differ in d’ than those in the general tips condition (W = 59,904, *p* = .807, *r* = 0.009). Post-hoc tests were corrected for multiple comparisons by the FDR method. In sum, results show that participants in the specific tips condition spent the most time rating headlines compared to general tips and control conditions.

## Difference Score (Not preregistered)

We conducted non-preregistered analyses using participant-level difference scores, calculated as belief in real headlines minus belief in AIVM headlines. An examination of residuals revealed that the data were not normally distributed, and thus we opted to conduct a Kruskal-Wallis test. Results revealed significant differences between conditions, χ^2^(2) = 7.88, *p* = .019, with median difference score of 0.70 in the specific tips condition, 0.55 in the general tips condition, and 0.45 in the control condition. Wilcox rank-sum post-hoc tests showed that participants in the specific tips condition had greater difference scores than those in the general tips (W = 54,168, *p* = .046, *r* = 0.082) and control conditions (W = 52,629, *p* = .009, *r* = 0.100). Difference scores did not significantly differ between participants in the control condition and those in the general tips condition (W = 59,216, *p* = .615, *r* = 0.019). Post-hoc tests were corrected for multiple comparisons by the FDR method. These results showed that participants in the specific tips condition showed a larger difference in beliefs between AIVM headlines and real headlines compared to those in the general tips and control conditions.

## Linear Mixed-models Analysis (Not preregistered)

We conducted linear mixed model analyses (Bates, 2005). The outcome variable was headline belief, and we included a fixed interaction term between condition (specific, general and control) and headline veracity (real, AIVM). Random effects of participant and headline were included. Because we were particularly interested in seeing how the specific tip condition compared to other conditions, we set the specific tip condition as the reference level.

We found that there was a significant positive interaction coefficient between the general tips condition and belief in AIVM headlines (*b* = 0.20, *p* < .001) and between the control condition and AIVM headlines (*b* = 0.23, *p* < .001). This suggested that compared to participants in the specific tip condition, participants in both the general tips and control conditions showed less difference in belief between AIVM and real headlines (i.e. decreased discernment). Post hoc tests revealed that for AIVM headlines, participants in the specific tips condition showed less belief in AIVM than those in the general tips condition with an estimate of 0.22 (z = 2.38, SE = 0.09, *p* = .017). Participants in the control condition had significantly higher belief than those in the general tips condition with an estimate of 0.50 (z = 5.55, SE = 0.09, *p* < .001), and had significantly higher belief than those the specific tips condition with an estimate of 0.72 (z = 7.91, SE = 0.09, *p* < .001). For real headlines, belief did not differ between participants in the specific tips condition and those in the general tips condition, with an estimate of 0.02 (z = 0.17, SE = 0.09, *p* = 0.862). Participants in the control condition had significantly higher belief in real headlines than those in the general tips condition with an estimate of 0.47 (z = 5.19, SE = 0.09, *p* < .001) and those in the specific tips condition with an estimate of 0.48 (z = 5.34, SE = 0.09, *p* < .001). These results suggest that when accounting for random effects of participants and headlines, specific tips exhibited higher discernment and lower belief in AIVM headlines compared to the general tips and control conditions, while belief in real headlines did not differ between specific and general tips. Post-hoc tests were corrected for multiple comparisons by the false discovery rate (FDR) method.

## Correlation between memory for specific tips and discernment (Not preregistered)

Within the specific tips condition, memory for tips was positively correlated with d’, *r*(341) = 0.243, *p* < .001, indicating that improved memory for tips was associated with greater discernment between real and AIVM headlines, as seen in Figure 3C. Consistently, memory for tips was positively correlated with the difference score, *r*(341) = 0.245, *p* < .001, indicating that the difference in beliefs between real and AIVM headlines was greater when more tips were remembered.

**Table S2**

*Fixed and random effects of a linear mixed model measuring headline belief*

|  | Fixed Effects | | | | | |
| --- | --- | --- | --- | --- | --- | --- |
|  | Est/Beta | | | SE | t | p |
| Intercept | 4.44 | | | 0.29 | 15.23 | < .001 |
| Control condition | 0.48 | | | 0.09 | 5.34 | < .001 |
| General condition | 0.02 | | | 0.09 | 0.17 | .862 |
| AIVM headlines | -0.77 | | | 0.40 | 1.90 | .065 |
|  |  | | | | | |
| Control condition X  AIVM headlines | 0.23 | | | 0.06 | 3.89 | < .001 |
| General condition X  AIVM headlines | 0.20 | | | 0.06 | 3.35 | < .001 |
|  | | | | Random Effects | | |
|  | | | Variance | | | S.D. |
| Subject (Intercept) | | | 1.11 | | | 1.05 |
| Headline (Intercept) | | | 1.61 | | | 1.27 |
| Model fit | | | | | | |
| R^2^ | | Marginal | | | Conditional | |
|  | | 0.02 | | | 0.32 | |
| Model equation: headline belief ~ 1 + condition*veracity + (1 \| subject) + (1 \| headline) | | | | | | |

*Note.* The specific tips condition and real headlines were used as the reference level.

Inspection of residuals from the model showed that the data satisfied the normality assumption, and multicollinearity was low in all fixed effects, with adjusted Generalized Variance Inflation Factors of 1.06, 1.00 and 1.06 for condition, veracity and the interaction respectively. Levene’s test for equality of variances was conducted to assess the homogeneity of variances. The assumption of homogeneity of variances was not violated, F(2, 41577) = 0.43, *p* = .652.

# Supplement D: Realism, evidence and image surprise

Belief in AIVM did not pass the threshold of significance when correlated with image surprise (*r*(18) = -0.437, p = .054), but was positively correlated with realism (*r*(18) = 0.581, p = .007), and not correlated with evidence strength (*r*(18) = 0.232, p = .324). Belief in real headlines was not correlated with image surprise (*r*(18) = 0.130, p = .586), positively correlated with image realism (*r*(18) = 0.528, p = .017), and positively correlated with evidence strength (r = 0.653, p = .002). The same pattern of results was observed in the full sample.

# Supplement E: Discernment Descriptives

## Table S3

*Descriptive statistics from signal detection analyses (attentive subgroup)*

| **Condition** | **Measure** | **Mean** | **S.D.** |
| --- | --- | --- | --- |
| Specific | Discernment | 0.43 | 0.61 |
|  | Response bias | 0.34 | 0.57 |
|  | Hit rate | 0.46 | 0.18 |
|  | False alarm rate | 0.33 | 0.21 |
| General | Discernment | 0.29 | 0.54 |
|  | Response bias | 0.27 | 0.56 |
|  | Hit rate | 0.46 | 0.18 |
|  | False alarm rate | 0.36 | 0.20 |
| Control | Discernment | 0.26 | 0.59 |
|  | Response bias | 0.06 | 0.55 |
|  | Hit rate | 0.53 | 0.17 |
|  | False alarm rate | 0.44 | 0.21 |

*Note*: Participants who failed the attention check were excluded in these descriptives.

## Table S4

*Descriptive statistics from signal detection analyses (full sample)*

| **Condition** | **Measure** | **Mean** | **S.D.** |
| --- | --- | --- | --- |
| Specific | Discernment | 0.43 | 0.62 |
|  | Response bias | 0.31 | 0.61 |
|  | Hit rate | 0.47 | 0.19 |
|  | False alarm rate | 0.34 | 0.22 |
| General | Discernment | 0.30 | 0.53 |
|  | Response bias | 0.26 | 0.57 |
|  | Hit rate | 0.47 | 0.18 |
|  | False alarm rate | 0.37 | 0.20 |
| Control | Discernment | 0.24 | 0.58 |
|  | Response bias | 0.05 | 0.57 |
|  | Hit rate | 0.53 | 0.18 |
|  | False alarm rate | 0.44 | 0.22 |

*Note*: Participants who failed the attention check were not excluded in these descriptives.

# Supplement F: Linear Mixed Models Analysis

## Table S5

*Fixed and random effects of a linear mixed model measuring headline belief*

|  | Fixed Effects | | | | | |
| --- | --- | --- | --- | --- | --- | --- |
|  | Est/Beta | | | SE | t | p |
| Intercept | 4.40 | | | 0.30 | 14.81 | < .001 |
| Control condition | 0.50 | | | 0.09 | 5.57 | < .001 |
| General condition | 0.02 | | | 0.09 | 0.28 | .783 |
| AIVM headlines | -0.78 | | | 0.41 | 1.90 | .065 |
|  |  | | | | | |
| Control condition X  AIVM headlines | 0.23 | | | 0.06 | 3.75 | < .001 |
| General condition X  AIVM headlines | 0.20 | | | 0.06 | 3.30 | < .001 |
|  | | | | Random Effects | | |
|  | | | Variance | | | S.D. |
| Subject (Intercept) | | | 1.06 | | | 1.03 |
| Headline (Intercept) | | | 1.68 | | | 1.30 |
| Model fit | | | | | | |
| R^2^ | | Marginal | | | Conditional | |
|  | | 0.02 | | | 0.32 | |
| Model equation: headline belief ~ 1 + condition*veracity + (1 \| subject) + (1 \| headline) | | | | | | |

*Note.* The specific tips condition and real headlines were used as the reference level.

Inspection of residuals from the model showed that the data satisfied the normality assumption, and multicollinearity was low in all fixed effects, with adjusted Generalized Variance Inflation Factors of 1.06, 1.00 and 1.06 for condition, veracity and the interaction respectively. Levene’s test for equality of variances was conducted to assess the homogeneity of variances. The assumption of homogeneity of variances was not violated, F(2, 40477) = 0.93, *p* = .396.
